# An Elusive Diagnosis: Case Reports of Secondary Hemophagocytic Lymphohistiocytosis and Review of Current Literature

**DOI:** 10.7759/cureus.4548

**Published:** 2019-04-26

**Authors:** Qiu J Tong, Manasi M Godbole, Nishit Biniwale, Saad Jamshed

**Affiliations:** 1 Internal Medicine, Rochester Regional Health, Rochester, USA; 2 Hematology / Oncology, Rochester Regional Health, Rochester, USA

**Keywords:** hlh, hemophagocytic lymphohistiocytosis, ebv, serum ferritin

## Abstract

Hemophagocytic lymphohistiocytosis (HLH) is a rare and serious hematologic disorder characterized by severe immune system dysregulation with a cytokine storm and histologic evidence of hemophagocytosis. It can be inherited or develop secondary to other diseases. We present three cases of secondary HLH in patients with distinct backgrounds. Our objective is to characterize the unique features of the disease, its underlying associations, treatment, and potential prognostic variables.

The first case was a 20-year-old male with a history of intravenous (IV) drug abuse who presented with multi-organ failure and septic shock. A diagnosis of HLH was suspected after finding a ferritin of >100,000 ng/mL and confirmed with bone marrow biopsy. Furthermore, the patient was found to have significant Epstein-Barr virus (EBV) viremia. He responded well to the HLH-94 protocol with the addition of rituximab and ganciclovir. The second case was a 50-year-old female with a history of human immunodeficiency virus (HIV) who presented with multi-organ failure and severe anemia. Ferritin was also significantly elevated and a bone marrow biopsy confirmed the diagnosis of HLH. She was started on HLH-94 protocol. Despite treatment, the patient expired due to worsening renal failure and shock. Her autopsy report also showed evidence of Hodgkin’s lymphoma. The third case was a 57-year-old male with a history of Crohn’s disease treated with infliximab and adalimumab, who presented with multi-organ failure and pancytopenia. A diagnosis of HLH was made based on clinical findings and later confirmed on bone marrow biopsy. He responded to HLH-94 protocol but experienced fatal gastrointestinal bleeding.

Patients presenting with HLH are often critically ill and deteriorate rapidly. The diagnosis is often challenging to establish due to its variable presentation and association with other pathologies. A moderate index of suspicion should be present for patients who have febrile illness with pancytopenia, multi-organ failure, high ferritin, and low fibrinogen levels. We discuss associations with viral infections, hematologic malignancies and immunosuppressive therapy. Treatment is directed at suppressing the immune response and for secondary HLH, addressing the underlying conditions, such as use of rituximab for EBV viremia and treatment of lymphoma.

## Introduction

Hemophagocytic lymphohistiocytosis (HLH) is a serious, rare, and difficult to diagnose hematologic disorder characterized by severe immune system dysregulation, leading to the overproduction of inflammatory cytokines, hyperinflammation, and histologic evidence of hemophagocytosis [1, 2]. It is frequently associated with multi-organ involvement and carries a poor prognosis [1]. HLH can be inherited as an autosomal recessive disorder or develop secondary to other pathologies. There is no age restriction, though it presents more commonly in older children and young adults [2]. We present three cases of secondary HLH in adult patients with distinct backgrounds. Our objective is to characterize the unique features of the disease, three different underlying associations, approach to treatment, and potential prognostic variables.

## Case presentation

Case 1

A 20-year-old male with a history of intravenous (IV) drug abuse presented with progressively worsening shortness of breath and one week of flu-like symptoms. He was febrile and hypoxic on presentation. Chest X-ray showed diffuse infiltrates and CT scan of the chest was concerning for septic emboli. No vegetations were seen on transthoracic echocardiography (TTE) or transesophageal echocardiography (TEE) and blood cultures yielded no growth. CT scan of the abdomen was significant for hepatosplenomegaly and laboratory findings revealed acute kidney injury (AKI), elevated liver enzymes, and pancytopenia. Tests for hepatitis B, C, and human immunodeficiency virus (HIV) were negative. The patient developed worsening hypoxic respiratory failure, hypotension, and fevers warranting transfer to the medical ICU for intubation, aggressive fluid resuscitation, vasopressor support, and broad-spectrum antibiotics. He also required multiple blood and cryoprecipitate transfusions for anemia and hypofibrinogenemia. The diagnosis of HLH was entertained and further workup revealed a ferritin level of 104,940 ng/mL and LDH of 4,099 U/L. HLH was confirmed on hospital day 10 with a bone marrow biopsy revealing hemophagocytic histiocytes. A quantitative Epstein-Barr virus polymerase chain reaction (EBV PCR) test revealed >1,000,000 copies of viral DNA/mL. The patient responded well to the HLH-94 protocol with methylprednisolone and bi-weekly etoposide. Rituximab and ganciclovir were added for EBV viremia. Due to worsening renal failure, continuous renal replacement therapy was initiated on day 10 of hospitalization for five days. After two weeks of management at our facility and another week at an outside hospital, he recovered and was discharged home with close follow-up. He has remained relapse free now for 13 months.

Case 2

A 50-year-old female with a history of HIV and non compliance with highly active antiretroviral therapy (HAART) presented with one month of confusion, fevers, fatigue, and night sweats. Laboratory findings on admission were significant for hemoglobin of 4.6 g/dL, lactate of 2.8, and INR of 3.9 with normal liver function tests (LFTs). Her CD4 count was 51/mm3, HIV viral load less than 20 and leukocyte count otherwise normal. Hemoglobin and hematocrit responded to blood transfusions but no source of bleeding was identified. The patient remained febrile and hypotensive despite coverage with piperacillin-tazobactam, vancomycin, trimethoprim-sulfamethoxazole and acyclovir. Cultures remained unrevealing but her course was complicated by Clostridium difficile infection on day 4. On day 6 of hospitalization, she was transferred to the medical ICU requiring vasopressors and was intubated for worsening encephalopathy. MRI of the brain at this time suggested HIV encephalitis and abdominal CT showed splenomegaly and multiple hypodense lesions in the liver and spleen. She developed progressive oliguric renal failure for which hemodialysis was initiated on hospital day 9. Testing for viral pathogens revealed 64,000 copies of EBV DNA per mL, and serum ferritin was elevated at 2,512 ng/mL. Her platelet counts also fell gradually to 17,000/uL from normal levels on admission. HLH was suspected but treatment was not initiated as she only met four of the eight criteria for diagnosis. A bone marrow biopsy resulted on day 11 of hospitalization, confirming the presence of hemophagocytic cells. She was immediately started on HLH-94 protocol with etoposide and dexamethasone. Despite treatment, the patient’s hypotension, acidosis, and renal failure worsened. She did not tolerate hemodialysis despite vasopressor support and the family agreed to withdraw care. The patient passed away on day 12. Her autopsy report also showed evidence of Hodgkin’s lymphoma in her liver and spleen, which was a new diagnosis.

Case 3

A 57-year-old male with a history of Crohn’s disease previously on tumor necrosis factor (TNF) inhibitor therapy presented to the hospital with multi-organ failure and pancytopenia. Additional laboratory findings revealed low fibrinogen of 43, elevated ferritin of 108,416 ng/mL, elevated liver enzymes, and hyperbilirubinemia. Viral testing was negative for EBV and HIV. Due to the development of worsening oliguric renal failure, dialysis was started on day 2 of hospitalization. The diagnosis of HLH was made on day 5 of hospitalization based on clinical findings and bone marrow biopsy confirming hemophagocytic histiocytes. HLH-94 protocol was initiated with etoposide and dexamethasone. His liver function panel, LDH and ferritin levels all improved dramatically with treatment. However, on hospital day 35, the patient developed a severe gastrointestinal (GI) bleed requiring multiple transfusions. Upper and lower endoscopies were unable to locate the source of bleeding. His renal function failed to recover throughout the hospitalization and he remained dialysis-dependent. Furthermore, the patient was later noted to have proximal intestine as the source of bleeding, but he was a poor surgical candidate and after a complicated course, on hospital day 41, his family decided to shift goals of care towards comfort measures. Despite exhibiting signs of recovery from HLH, the patient passed away on hospital day 49 from complications of hemorrhagic shock.

## Discussion

The annual incidence of primary HLH is estimated to be approximately 1.2 per 1 million individuals [3]. Data on the incidence of secondary HLH is sparse, but it is thought to be more common [2]. Even with a well-defined diagnostic criterion, HLH is difficult to diagnose because of its variable presentation and association with other diseases. In cases like the ones we presented, the diagnosis is not made until days to weeks after admission. Common triggers leading to HLH include hematologic malignancy, infections, immunosuppressed state or autoimmune disease.

In younger individuals, secondary HLH is most associated with an infectious etiology. EBV is a well-recognized culprit, but there are also established associations with other viral (cytomegalovirus, HHV8, HIV, and parvovirus), bacterial (mycobacteria and mycoplasma), parasitic (leishmania and plasmodium), and fungal (candida and cryptococcus) infections [4]. In older populations, HLH most commonly develops secondary to hematologic malignancies such as leukemias or lymphomas, and rarely solid tumors [5, 6].

A number of autoimmune disorders have been associated with HLH as well. Rheumatologic diseases have been linked to HLH-like syndromes grouped together as 'macrophage activation syndromes' (MAS). Among these, systemic juvenile inflammatory arthritis (sJIA) is most frequently implicated, followed by systemic lupus erythematosus, Still’s disease, rheumatoid arthritis, Kawasaki disease, dermatomyositis, polyarteritis nodosa, sarcoidosis, and Sjögren’s syndrome [7, 8]. Rarely, patients on immunosuppressive therapy for autoimmune conditions can also develop HLH [7, 9]. Thiopurine treatment for inflammatory bowel disease is associated with an increased risk for developing viral infection driven HLH, and this risk is slightly higher in patients with Crohn’s disease versus ulcerative colitis [10-12].

The diagnostic criteria to suspect HLH are characterized by finding five of the following eight findings [1, 13].

1) Fever ≥38.5°C 2) Splenomegaly 3) Peripheral blood cytopenias with at least two of the following: - Hemoglobin <9 g/dL - Platelets <100,000/µL - Absolute neutrophil count <1000/µL 4) Either fasting hypertriglyceridemia (>265 mg/dL) or hypofibrinogenemia (<150 mg/dL) 5) Hemophagocytic lymphocytes in bone marrow, spleen, lymph node, or liver 6) Low or absent NK cell activity 7) Ferritin >500 ng/mL 8) Elevated soluble CD25 (alpha-chain of the IL-2 receptor) >2400 U/mL

While obtaining a bone marrow biopsy is important, its sensitivity can be as low as 60% [14]. Treatment should not be delayed if biopsy is negative and the patient otherwise meets five of the above diagnostic criteria [14, 15]. The cutoff for ferritin has been another source of contention, as it is an acute-phase reactant that can be elevated in many differential diagnoses [13]. One study found that a ferritin level greater than 10,000 ug/L corresponds to a 90% sensitivity and 96% specificity for HLH [13]. Elevated soluble CD25 levels are more specific for the diagnosis, but can take several days to result [15]. For primary HLH, molecular genetic testing is available to further classify disease subtype. Common gene mutations involved in heritable HLH include PRF1, UNC13D, STX11, and STXBP2 [16]. Testing can also be done for mutations in RAB27A for Griscelli syndrome and CHS1 for Chediak-Higashi syndrome, both of which are associated with the development of HLH [16].

When it comes to treating these patients, the immediate goal is immunosuppression to inhibit the physiological pathways leading to hyperinflammation. HLH-94 regimen combines etoposide with high-dose dexamethasone to achieve immunosuppression. The HLH-2004 regimen is more intensive, adding cyclosporin A, methotrexate, and hydrocortisone. Patients with primary HLH should receive the same treatment, but they ultimately require hematopoietic stem cell transplant. For patients with EBV, adding rituximab can potentially improve outcomes by hypothetically reducing B cells infected by the virus [17]. Intravenous immunoglobulin (IVIG), anti-TNF agents, and IL-1 inhibitors are other medications that can be efficacious in certain cases [18]. Response to treatment should be monitored closely with basic laboratory tests and based on clinical exam. The underlying medical condition, whether it is infection, autoimmune disease or malignancy, needs to be addressed as well to prevent recurrence of HLH [14].

Prognostic indicators are not well established due to the relatively uncommon occurrence of HLH. It is thought that the degree of inflammation and end-organ damage correlates with higher mortality, but other variables such as patient age and comorbidities invariably affect outcomes. Secondary hemophagocytic syndrome is fatal in 22%-59% of patients [14]. In particular, HLH associated with active EBV disease is known for its aggressive progression and poor prognosis, in which EBV viral load correlates with decreased survival [19]. Having an underlying malignancy is also associated with rapid disease progression. One study found that patients with tumor-associated HLH had a median overall survival of less than two months, significantly less than non-tumor-associated HLH in which median survival was close to two years [5].

## Conclusions

Patients presenting with secondary HLH tend to be critically ill and deteriorate rapidly. A reasonable index of suspicion should always be present for any patient who has febrile illness with pancytopenia, high ferritin, and low fibrinogen levels. More common causes should always be ruled out, but treatment should not be delayed if the diagnosis is suspected. As long as patients meet five of the eight diagnostic criteria, a positive bone biopsy is not necessary to justify treatment. Complications such as sepsis, bleeding, and multi-organ failure commonly lead to dismal outcomes. Although the prognosis is quite poor overall, HLH is treatable as demonstrated in two of the cases we presented. Current treatment guidelines recommend the use of etoposide and steroids. Evidence also supports a potential benefit from the addition of rituximab when there is EBV viremia. Underlying medical conditions that trigger HLH also must be addressed.
